# Functional Syndesmotic Widening: A Case Report and Literature Review

**DOI:** 10.7759/cureus.63531

**Published:** 2024-06-30

**Authors:** Samuel Sing Li Ong, Ernest Beng Kee Kwek

**Affiliations:** 1 Department of Orthopaedic Surgery, Sengkang General Hospital, Singapore, SGP; 2 Department of Orthopaedic Surgery, Woodlands Health, Singapore, SGP

**Keywords:** tibiofibular overlap, syndesmosis injury, high ankle sprain, ankle sprain, functional syndesmotic widening

## Abstract

Injury to the distal tibiofibular syndesmosis can be a diagnostic challenge in the absence of advanced imaging. We report a case of a 21-year-old male patient who sustained an ankle injury and demonstrated radiological evidence of syndesmosis widening on plain radiographs. He underwent endobutton fixation which resulted in anterior subluxation of the talus and difficulty in ankle dorsiflexion. This is the first case report in the literature of a functional syndesmotic widening and the subsequent sequelae when subjected to a stabilisation procedure. The previously reported risk factors were inconsistent with our patient’s demographics of a young, previously obese adult. We postulate that his childhood morbid obesity likely contributed to the functional widening of his ankle syndesmosis.

## Introduction

An isolated syndesmosis injury, more commonly referred to as a high ankle sprain, is a traumatic ligamentous injury to the distal tibiofibular syndesmosis. Even in the hands of the most experienced orthopaedic surgeon, its diagnosis still remains a challenge, especially in the absence of advanced imaging. The distal tibiofibular syndesmosis is a fibrous joint where the concave distal lateral tibia (incisura tibialis) articulates with the convex distal medial fibula. It comprises the anterior-inferior tibiofibular ligament (AITFL), posterior-inferior tibiofibular ligament (PITFL), interosseous ligament (IOL), inferior transverse ligament (ITL) and interosseous membrane (IOM) [1]. These strong ligaments and membranes work in tandem to provide proximal stability to the talo-crural joint, resisting both rotational and translational forces. A cadaveric study demonstrated the contribution of each individual ligament towards stability: AITFL (35%), deep PITFL (33%), superficial PITFL (9%) and IOL (22%) [2].

There are a few provocative tests to clinically evaluate for a syndesmosis injury which includes the squeeze test (Hopkin’s), external rotation stress test (Kleiger’s), fibular translation test and cross-leg test [3]. However, these tend not to be very reliable. de César et al. reported a 30% sensitivity and 93.5% specificity for Hopkin’s, while a 20% sensitivity and 84.8% specificity for Kleiger’s [4]. For ankle fractures, a Cotton’s test is performed intra-operatively after bony fixation to assess for syndesmosis integrity. This is done by applying a lateral directed force on the distal fibula with a bone hook, looking out for any opening of the distal tibiofibular joint.

Plain radiographs of the ankle are the first line of imaging to assess for syndesmosis injury. Harper and Keller posited normal syndesmotic parameters after studying 12 cadavers based on a 95% confidence interval [5]. Firstly, a tibiofibular clear space (TFCS) of <6mm on anteroposterior (AP) or mortise views. Secondly, a tibiofibular overlap (TFO) of >6mm on AP view. Thirdly, a TFO >1mm on mortise view. Additionally, an increased medial clear space is also suggestive of a syndesmosis injury. There is also role for advanced imaging modalities such as ultrasound, CT or MRI to evaluate suspected syndesmosis injuries. Surgical intervention for an unstable syndesmosis injury is recommended to reduce the syndesmosis and to minimise the risk of developing secondary osteoarthritis of the ankle joint. Syndesmosis stabilisation in a high ankle sprain can be performed with either syndesmotic screws or endobuttons.

## Case presentation

**Figure 1:**
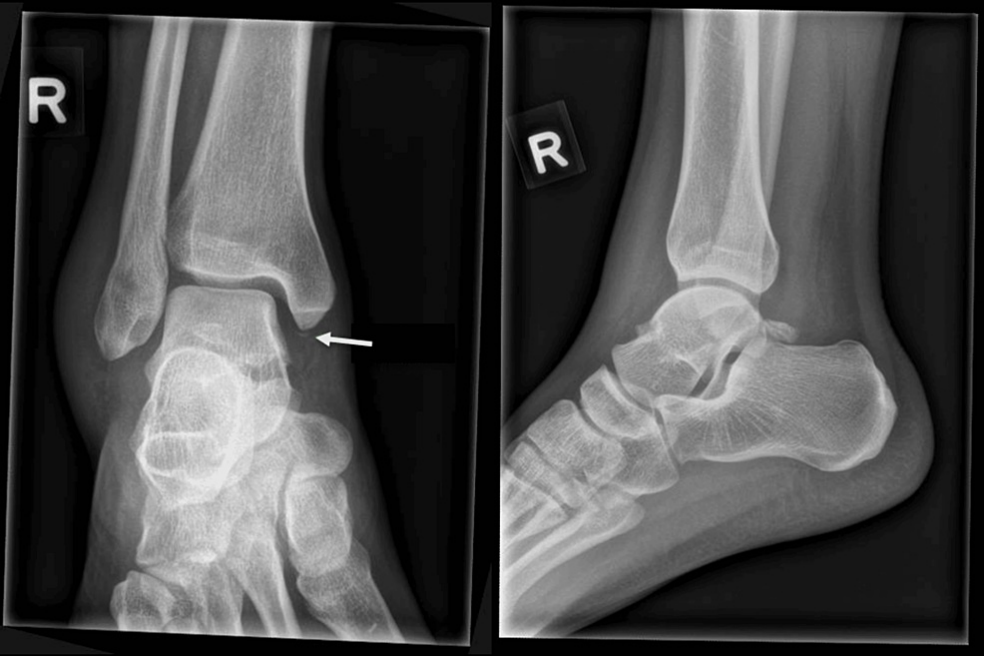
Initial radiographs of the right ankle showed a small avulsion fracture at the tip of the medial malleolus (arrow), swelling most prominent at the lateral aspect and suggestion of widening of syndesmosis.

A 21-year-old male student, with no known past medical history, first presented to our institution’s emergency department after suffering an injury to his right ankle while playing badminton that morning. He complained of a painful and swollen ankle. Upon examination, bimalleolar swelling was noted with exquisite tenderness at the medial malleolus. Non-weightbearing ankle radiographs (Figure 1) showed a small avulsion fracture at the tip of the medial malleolus. He was placed on a posterior short-leg splint and given an early outpatient appointment with an orthopaedic specialist.

**Figure 2:**
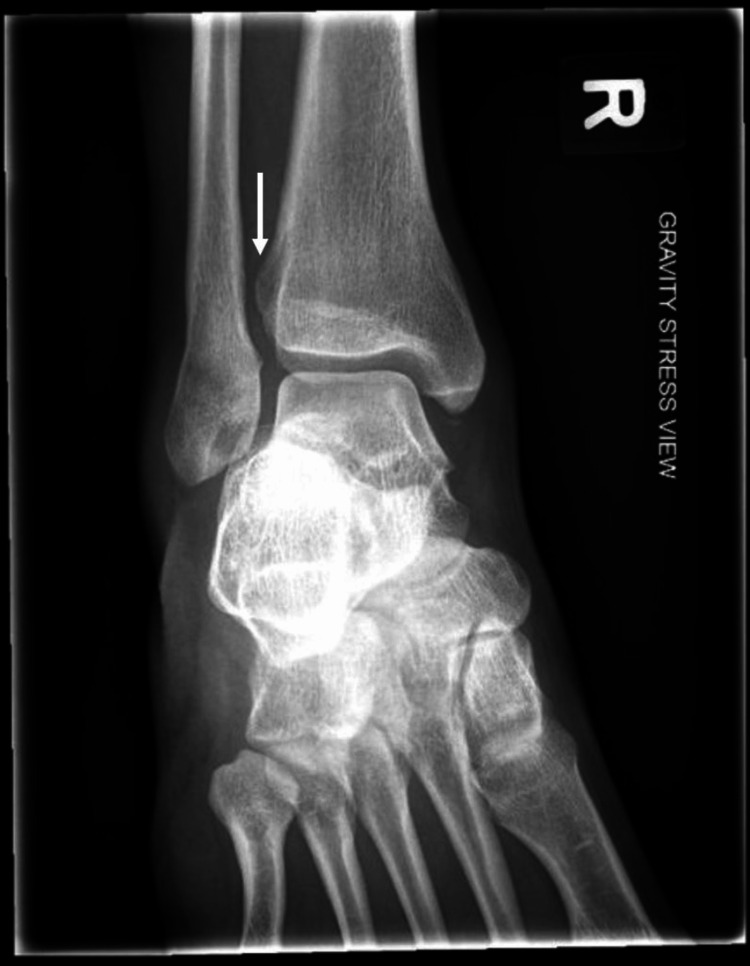
Gravity stress view showing marked TFCS widening of 7.21mm and no TFO (arrow). TFCS: tibiofibular clear space; TFO: tibiofibular overlap.

The patient was subsequently reviewed in the outpatient setting by an orthopaedic trauma specialist. He gave a history of multiple ankle sprains with the latest being an inversion injury. Clinically, there was generalised moderate swelling. On palpation, there was minimal medial sided pain, the proximal fibula was non-tender and tenderness was localised to the distal fibula. A gravity stress radiograph (Figure 2) revealed marked TFCS widening without TFO. The patient was diagnosed with a right high ankle sprain and was counselled for endobutton fixation.

**Figure 3:**
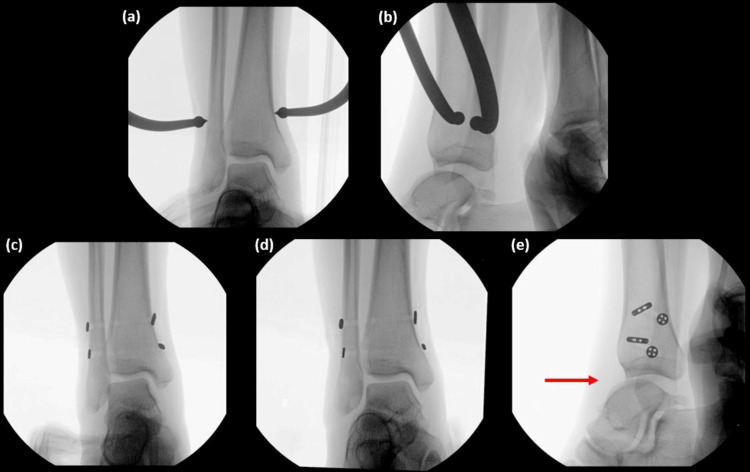
Intra-operative fluoroscopy images. AP (a) and lateral (b) views of syndesmotic reduction after clamping. AP (c), mortise (d) and lateral (e) views after endobutton fixation, showing good syndesmotic reduction but anterior subluxation of the talus (arrow). AP: anterior-posterior.

During the procedure, the syndesmosis was clamped and two endobuttons were passed posterolateral to anteromedial. Intra-operative fluoroscopy (Figures 3a-3e) showed satisfactory reduction of the distal tibiofibular joint, but the talus was noted to be subluxed anteriorly. Initially, we assumed it was due to mal-reduction, but recheck fluoroscopy confirmed that normal syndesmotic parameters were restored post-reduction. We also considered performing an ankle arthroscopy for direct visualisation of the syndesmosis, but the decision was made to complete the surgery as consent was only obtained for syndesmotic stabilisation.

**Figure 4:**
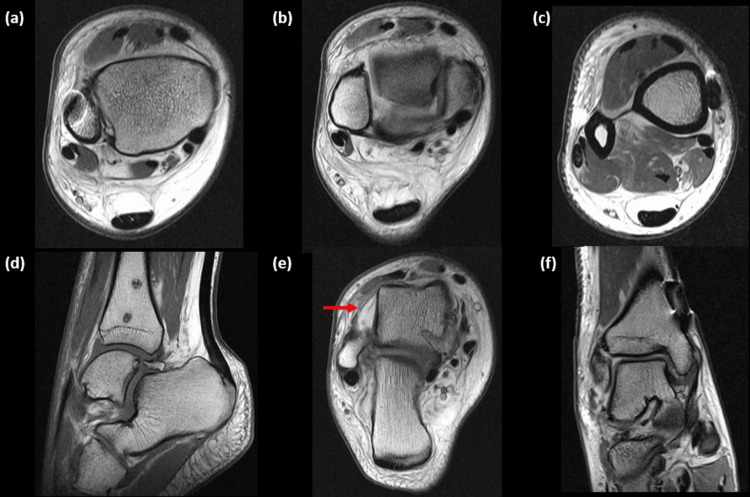
MRI of the right ankle after syndesmotic fixation was performed for further evaluation. Syndesmosis was intact: AITFL (a), PITFL (b) and IOL (c). Known anterior subluxation of talus (d). ATFL complete tear (e, arrow), CFL partial-thickness tear and PTFL sprain. Partial-thickness tear of the deep deltoid (talo-tibial) ligament and sprain/partial-thickness tear of the superficial deltoid ligamentous component (f). MRI: magnetic resonance imaging; AITFL: anterior-inferior tibiofibular ligament; PITFL: posterior-inferior tibiofibular ligament; IOL: interosseous ligament; ATFL: anterior talo-fibular ligament; CFL: calcaneofibular ligament; PTFL: posterior talo-fibular ligament.

Post-operatively day 1, the patient experienced difficulty in dorsiflexion; thus, an MRI scan was organised. The syndesmosis was intact, AITFL (Figure 4a), PITFL (Figure 4b) and IOL (Figure 4c). His main injury was an anterior talo-fibular ligament (ATFL) complete tear (Figure 4e), along with the known medial malleolar avulsion fracture which was associated with a partial-thickness tear of the deep deltoid ligament (Figure 4f). At the level of the distal endobutton, asymmetry of the tibiofibular interval was noted, measuring 2mm anteriorly and 4.8mm posteriorly. The fibula was 13.4° internally rotated, and anterior subluxation of the talus was evident (Figure 4d).

Post-operatively day 2, further history was elicited from the patient. He had multiple bilateral ankle sprains with recent episodes being worse. He had a history of childhood obesity, weighing 100kg at age 12 but had slimmed down to 71kg currently. He had no clinical evidence of generalised joint hyperlaxity. The patient was thereafter consented for removal of endobuttons under anaesthesia.

Post-operatively day 3, the patient underwent removal procedure. Contralateral intra-operative fluoroscopy revealed similar findings, widened TFCS, minimal overlap and widened syndesmosis of his left ankle. Pre-removal, the right talus was in the mortise, anteriorly subluxed but not irreducible. Post-removal, the syndesmosis widened back to the pre-operative magnitude, but the talus was fully reduced and his ankle could dorsiflex to 10°.

**Figure 5:**
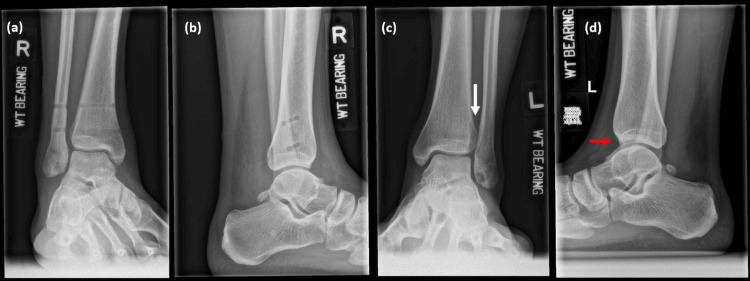
Weightbearing ankle radiographs performed two weeks after removal of endobuttons. Right AP (a) and lateral (b) weightbearing ankle radiographs performed two weeks after removal of endobuttons showed his talus was reduced and the syndesmosis was restored to pre-operative magnitude. Contralateral AP (c) and lateral (d) views of his normal unoperated left ankle also showed anterior subluxation of his talus (red arrow) and syndesmotic widening (white arrow). AP: anterior-posterior.

Orthogonal weightbearing radiographs were performed two weeks after discharge which demonstrated his normal left ankle also had evidence of syndesmotic widening (Figures 5a-5d). He was progressed to full weightbearing status and was referred to the physiotherapist for rehabilitation to treat as for an ATFL sprain.

Two months post-operation, the patient was recovering well. He could walk with no complaints and had started jogging. His right ankle also regained full range of motion.

## Discussion

This is the first case report in the literature of a functional syndesmotic widening and the subsequent sequelae when subjected to a stabilisation procedure. We initially postulated that the anterior subluxation of the talus after syndesmotic fixation was due to mal-reduction. Anatomically, the trapezoidal-shaped talus is narrower posteriorly; thus, overtightening of the syndesmosis will result in the talus translating anteriorly to allow the talar body to reside in the mortise. Hoshino et al. reviewed 364 ankle fractures with syndesmotic screw fixation, of which three had mal-reduction of the tibiotalar joint in the sagittal plane post-operatively [6]. However, these were due to avoidable technical errors. In our case, this was checked multiple times intra-operatively and it was confirmed that normal syndesmotic parameters were restored post-reduction.

A functional syndesmotic widening can be defined as an intact syndesmosis despite evidence of an increased TFCS and/or a decreased TFO on plain radiographs. During our patient’s presentation with an acute ATFL injury, the decision to proceed with syndesmotic stabilisation was confounded by the radiological appearance of a high ankle sprain. Having reduced the natively widened syndesmosis, this then tightened the mortise inadvertently and resulted in the subluxation of the talus anteriorly from the mortise and limiting ankle dorsiflexion. Restoring the functional space by removing the endobuttons thus alleviated his symptoms.

Since there is a lack of a ‘gold standard’ test to diagnose a syndesmosis injury, radiological parameters previously established by Harper and Keller are still widely accepted [5]. Its sensitivity, specificity and accuracy on AP radiographs are 44.1%, 100% and 63.5%, respectively, while 58.3%, 100% and 71.2%, respectively, on mortise radiographs [7].

Advanced imaging can be considered to aid diagnosis. Although ultrasound imaging is more accessible and produces dynamic images, it is operator dependent and reported to have a 66% sensitivity, 91% specificity, 86% positive predictive value and 77% negative predictive value for AITFL rupture [8]. For MRI, sensitivity, specificity and accuracy of diagnosing, an AITFL tear was 100%, 93.1% and 96.2%, respectively, and 100%, 100% and 100% for a PITFL tear [7].

Diagnostic ankle arthroscopy can be performed intra-operatively to obtain direct visualisation of the syndesmosis before definitive fixation. However, we do acknowledge that not all orthopaedic surgeons have the skillset or equipment available to accurately perform such a specialised procedure.

There have been limited reports in the literature describing syndesmotic widening as a normal phenomenon. Shah et al. reported that no TFO on the mortise view may represent a normal variant after analysing 392 healthy ankles [9]. Sowman et al. obtained CT images from 324 patients for reasons other than ankle pathology and four patients (1.23%) had no TFO [10]. The mean age of these four were 61.8, and they had no previous ankle pain, surgery or trauma.

Mei-Dan et al. evaluated 110 healthy individuals with dynamic ultrasound revealed that TFCS decreases with age and females had a greater functional widening [11]. There was no correlation with activity, height or length. For paediatrics, Bozic et al. reported 23% of injury-free children (aged between one and 15) had TFCS ≥6mm [12]. TFO only started to appear on the AP view at age five. On the mortise view, girls began to show overlap from age 10 compared to 16 for boys. Eighty-three percent had TFO ≤6mm on AP view compared to 85% with TFO ≤1mm on mortise view [12].

However, the risk factors identified above were inconsistent with our case study (a 21-year-old male). We postulate that his childhood morbid obesity likely contributed to his functional widening of both ankle syndesmosis as part of his development and persisted despite his weight loss. This could be due to the direct mechanical stress placed on the syndesmosis or the indirect action of excessive oestrogen secondary to increased adiposity resulting in syndesmotic laxity. This mechanism has heretofore not been described in the literature.

## Conclusions

In conclusion, this case report highlights that a functional syndesmotic widening can exist and surgeons should be aware of this phenomenon. Ultimately, the diagnosis of a distal tibiofibular syndesmosis injury based on numerical parameters on plain radiographs may be insufficient and continues to remain a challenge. We recommend a holistic approach to diagnose a distal tibiofibular syndesmosis injury, with the knowledge that a functional syndesmotic widening can exist. If the diagnosis still remains in doubt, advanced imaging modalities can be considered pre-operatively or an intra-operative diagnostic ankle arthroscopy can be performed.
